# Epidemiology and Impact of Staff Training in Controlling Complications Related to Central Venous Catheters in 4 Intensive Care Units in a French University Hospital: Protocol of a Prospective, Quasi-Experimental, Before-After Study

**DOI:** 10.2196/74985

**Published:** 2025-07-23

**Authors:** Nagham Khanafer, Anne-Claire Lukaszewicz, Elisabetta Kuczewski, Laurent Argaud, Philippe Vanhems

**Affiliations:** 1 Department of Hygiene, Epidemiology, and Prevention Edouard Herriot Hospital Hospices Civils de Lyon Lyon France; 2 International Center for Infectiology Research (CIRI), Inserm U1111, CNRS UMR5308, ENS de Lyon Lyon 1 University Lyon France; 3 Anesthesia and Intensive Care Unit Edouard Herriot Hospital Hospices Civils de Lyon Lyon France; 4 Medical Intensive Care Unit Edoaurd Herriot Hospital Hospices Civils de Lyon Lyon France

**Keywords:** audit, central venous catheter, complications, education, guidelines, practices, training

## Abstract

**Background:**

Central venous catheters (CVCs) are essential tools in the management of critically ill patients in intensive care units (ICUs). However, their use is associated with several preventable complications, including central line–associated bloodstream infections (CLABSIs), catheter-related thrombosis, and mechanical events (eg, occlusion and rupture). Despite the availability of evidence-based guidelines from the French Society of Hospital Hygiene for CVC insertion and maintenance, adherence variability persists across health care settings, contributing to prolonged hospitalization, increased morbidity and mortality, and even increased health care costs. In 2022, an audit conducted at Edouard Herriot Hospital (Hospices Civils de Lyon, Lyon, France) revealed a degree of variation in adherence to current protocols. Specific deficits were noted in practices such as hand hygiene, flushing procedures, and aseptic techniques, among others.

**Objective:**

This study aims to (1) evaluate the effectiveness of a multifaceted educational intervention in improving health care workers’ adherence to national CVC management guidelines; (2) assess changes in complication rates (CLABSIs, thrombosis, and mechanical events) and patient outcomes (readmissions and mortality); and (3) identify factors associated with CVC-related complications through multivariate analysis.

**Methods:**

We are conducting a prospective, quasi-experimental, before-after study in 4 adult ICUs at a French university hospital. The protocol includes preintervention and postintervention audits using a digital tool to capture real-time compliance data, as well as standardized educational sessions based on French Society of Hospital Hygiene recommendations. The intervention consists of face-to-face training, posters, and digital reminders, all delivered by the same team to ensure consistency. Clinical data include demographics, comorbidities, CVC type and duration of use, administered medications, and complications. All patients with CVCs, including centrally inserted catheters (jugular or subclavian), femoral lines, and peripherally inserted central catheters, are eligible. The primary outcome is the incidence rate of CVC-related complications per 1000 catheter-days. Secondary outcomes include rates of hospital readmission, ICU and in-hospital mortality, and the degree of improvement in guideline adherence across participating units.

**Results:**

As of submission, 640 patients have been included in the study. Data collection remains ongoing. Data entry and final statistical analyses are expected to be completed in the third quarter of 2025.

**Conclusions:**

By combining audits, education, and feedback, this study will provide key insights into the effectiveness of education-based interventions for improving CVC-related practices in critical care settings. It may serve as a scalable model for enhancing patient safety and care quality in ICU settings. Results will be disseminated through peer-reviewed publications and international conferences.

**International Registered Report Identifier (IRRID):**

DERR1-10.2196/74985

## Introduction

Central venous catheters (CVCs) are essential tools in the care of critically ill patients in intensive care units (ICUs), providing vital vascular access for hemodynamic monitoring, administration of medications, and nutritional support. Despite their significant benefits, their use is associated with significant risks, including mechanical issues (eg, occlusion, rupture, and malposition), catheter-related thrombosis, and infectious complications such as central line-associated bloodstream infection (CLABSI). Among these, CLABSIs remain a persistent and significant concern for health care workers (HCWs) in ICUs, contributing to prolonged hospital stays, increased health care costs, and higher mortality rates [1].

Over the past decade, substantial progress has been made in reducing CLABSI rates through evidence-based guidelines and prevention bundles—including proper hand hygiene, full barrier precautions during insertion, use of alcohol-based antiseptics, optimal site selection, prevention of port line occlusions, and daily reassessment of catheter necessity [2,3]. However, despite these advancements, CLABSI continues to pose a serious concern. Its incidence varies depending on patient populations and health care settings, with estimates ranging from 0.5 to 5 per 1000 catheter-days [4]. In our institution, surveillance data collected between 2017 and 2022 show a mean CLABSI incidence of 3.11 per 1000 CVC-days. A peak was observed in 2021, reaching 5.1 CLABSIs per 1000 CVC-days, likely reflecting the impact of the COVID-19 pandemic. Although our average rate is lower than reported in a recent multinational, prospective study (4.82 CLABSIs per 1000 CVC-days) [5]. This variability underlines the need for targeted interventions to reinforce guideline adherence and reduce preventable complications.

Clinical guidelines are intended to support HCWs in delivering evidence-based care, but implementation is often inconsistent. The growing volume of recommendations, coupled with the complexity of ICU care and the evolving severity of patient conditions, complicates adherence. Furthermore, variability in practice both within and between institutions can significantly impact care quality. To better understand local practices, an audit was conducted in 2022 at Edouard Herriot Hospital (Hospices Civils de Lyon, Lyon, France) in collaboration with Becton, Dickinson and Company (BD). This audit revealed variability in adherence to established CVC management protocols. While over 70% of observations followed recommended practices—such as the use of gauze and alcohol-based antiseptics for line handling and appropriate catheter flushing—key areas, such as hand hygiene, flushing techniques, and aseptic measures, were suboptimal [6]. These findings underscored the need for targeted interventions to improve compliance.

Educational interventions have been shown to enhance HCWs’ knowledge, improve practices, and reduce complication rates [7,8]. Combining audits with targeted training can help bridge the gap between knowledge and bedside practice [9]. Moreover, bundling key evidence-based guidelines into practical, point-of-care tools has proven effective in improving adherence and reducing preventable complications [10].

In response to the audit findings, we developed a structured intervention that integrates audits and education to improve professional competencies and reduce the incidence of CVC-related complications.

The primary objective of this study is to evaluate the impact of an educational program on adherence to national CVC management recommendations issued by the French Society of Hospital Hygiene (SF2H). Secondary objectives include assessing whether improved adherence results in reduced CVC-related complications (including CLABSI, catheter-related thrombosis mechanical complications, readmissions related to CVC use, and associated mortality), and identifying risk factors associated with such complications, taking into account both clinical variables and observed care practices.

## Methods

### Ethical Considerations

The study participants’ personal information will be gathered and handled exclusively on the legal basis established by the applicable regulations, in line with Lyon University hospitals’ public service missions. Participants are provided with an information letter outlining the study’s objectives and follow-up procedures.

The research protocol was approved by the Institutional Ethical Board (reference number 2024 06 27 20). Additionally, the Comité National de l’Informatique et des Libertés of Hospices Civils de Lyon has granted approval for the study under reference number 23-115.

### Study Site

The study is being conducted in the 4 ICU wards of Edouard Herriot Hospital, a university hospital in Lyon, France.

### Study Design

**Figure 1:**
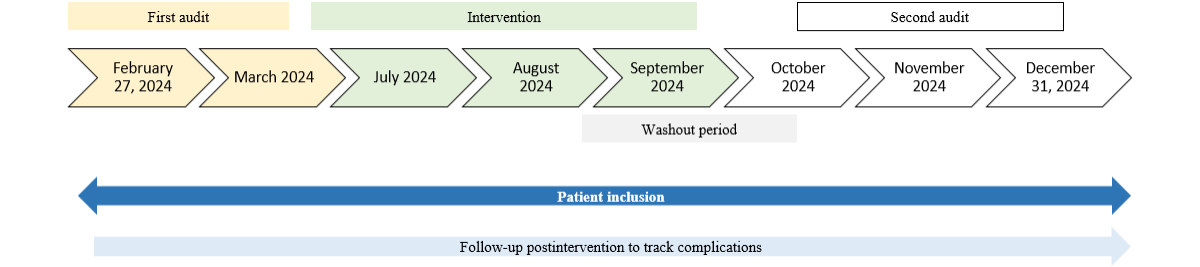
Study design of a prospective before-after study conducted in the 4 intensive care units of Edouard Herriot Hospital, Lyon, France. Audits were performed using Becton Dickinson Signature Solutions. The intervention was an educational program. The second audit was completed after a washout period of 2-3 months following the last session of the educational intervention. The follow-up was 30 days per central venous catheter to track complications (eg, central line–associated bloodstream infections, catheter-related thrombosis, mechanical damage, readmission for central venous catheter–related complications, and related death).

This is a prospective, quasi-experimental, before-after study combining audits, interventions, and clinical data. Figure 1 summarizes the study stages and timeline.

All consecutive patients with a CVC aged 18 years or older, hospitalized for at least 48 hours in 1 of the 4 ICU units of the hospital, and affiliated with the French national health insurance system were eligible. Recruitment occurred between February 27 and December 31, 2024. Patients with hemodynamic instability, receiving palliative care, or unable to engage in the information process independently or via a representative were excluded.

For this study, CVC refers to all central venous access devices, including centrally inserted central catheters (which may be jugular or subclavian), femoral inserted central catheters, and peripherally inserted central catheters, as per nomenclature endorsed by the World Congress on Vascular Access/Global Vascular Access Network and the European Society of Anesthesiology [11,12].

### Study Procedures

#### Audits

**Figure 2:**
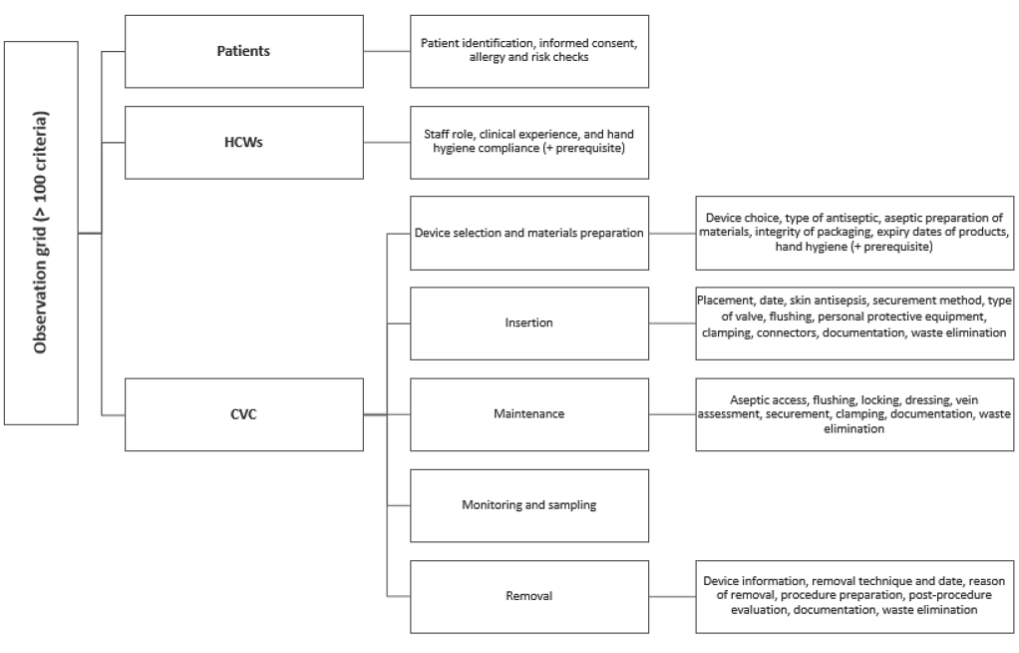
Structure of the observation framework used in the study. The audit tool followed the guidelines set by the French Society of Hospital Hygiene. CVC: central venous catheter; HCW: health care worker.

A standardized auditing tool was developed based on SF2H recommendations [13-15]. The questionnaire includes approximately 100 items covering hand hygiene; skin antisepsis; CVC insertion, maintenance, and removal; and documentation. It also collects data on HCWs’ professional education and clinical experience, which are considered potential factors influencing adherence to recommended practices. The main structure of the observation framework is detailed in Figure 2.

Audit observations were recorded using Signature Solutions, a digital tool developed by BD which captures real-time bedside practices [6]. Observations were recorded offline via secure tablets and uploaded for analysis by the hospital team.

Two audits were planned in this study: 1 before and 1 after the educational intervention. All analyses will be conducted independently by the research team without any BD involvement to maintain objectivity and scientific integrity.

#### Intervention (Educational Program)

The intervention included face-to-face exchange sessions, departmental poster displays, and electronic communications. Content focused on essential aspects of CVC management (eg, insertion procedures, drug administration, line handling, verification of reflux and tract patency, and timely removal of unnecessary catheters). Infection prevention measures were emphasized, including proper hand hygiene, disinfection protocols (including the type and formulation of antiseptic), vascular line handling, flushing techniques (including syringe type, volume, and method), use of personal protective equipment, and the application of full barrier precautions.

Training sessions were standardized and delivered by the same team across all ICUs to ensure consistency. Posters summarizing key practices were displayed uniformly in all ICUs, and digital reminders were sent via institutional communication channels.

To clarify the contents and objectives of this intervention, Table 1 summarizes the key points of the SF2H recommendations that were integrated into the training.

**Table 1:** Summary of French Society of Hospital Hygiene (SF2H) recommendations integrated into the educational intervention [14,15].

Theme	Main recommendations from the SF2H
Hand hygiene	Perform hand hygiene using alcohol-based hand rub before and after all catheter-related procedures.
Skin antisepsis	Use alcohol-based chlorhexidine (2%) for skin disinfection before insertion and during dressing changes.
Insertion technique	Apply barrier precautions.
Flushing procedures	Use single-use syringes; flush with sterile saline (minimum 10 mL) using the pulsating technique.
Manipulation of lines	Disinfect catheter hubs with alcohol-based antiseptic before any access.
Dressing and maintenance	Use sterile transparent dressings; change every 7 days or sooner if soiled or detached.
Catheter removal	Remove as soon as it is no longer clinically necessary (daily reassessment required).

#### Data Collection

Following verification of inclusion criteria, enrollment occurred upon CVC insertion or upon ICU admission for patients with preexisting inserted CVCs. A standardized questionnaire captured demographics, comorbidities as defined by *International Classification of Diseases, Tenth Revision* codes, information on hospital stay, total number of catheters on admission and during the stay in the ICU, products administered by CVC, CVC details (type, date and site of insertion, dressing, securement, and connectors). Data on antibiotics (molecule and duration) will be fully collected. Patients were monitored until discharge, death, or withdrawal. Those discharged with a CVC were contacted every 15 days until CVC removal or the conclusion of the study to determine whether complications occurred in the interim within 30 days of inclusion. No additional testing was requested for the purpose of this study.

Data collection is ongoing and will be completed during the second quarter of 2025.

#### Data Entry

Clinical data were recorded on paper in specific clinical record folders. Epi Info (US Centers for Disease Control and Prevention) will be used to create a digital survey for data entry. Different tables with data obtained upon admission, during the hospital stay, or at follow-up will be created. For each table, a unique key will be used to link with the main questionnaire. Data entry was planned for June 2025.

#### Data Management and Handling of Missing Data

All data will be reviewed for completeness and consistency. In the case of incomplete data, records with missing fields will be reported. Audit data had minimal missingness due to real-time entry. In contrast, loss to follow-up may occur in the clinical cohort, particularly among patients discharged with a CVC. These cases will be recorded, and reasons for the loss to follow-up will be documented when available. If the rate of missing data is nonnegligible, sensitivity analyses will be conducted to evaluate the potential impact on outcomes.

#### Data Curation

All data will be stored in the hospital’s information systems and will be accessible exclusively to investigators. No data will be shared with BD. Data retention complies with French regulations.

### Outcomes

#### Primary Clinical Outcome

The primary outcome is the incidence of CVC-related complications before and after the educational intervention. The overall complication rate is the number of associated complications per catheter-day that occurred during its use.

Complications are defined as follows:

- CLABSI is defined as a laboratory-confirmed bloodstream infection occurring in a patient who had a CVC in place for at least 48 hours prior to the onset of infection, with no other apparent source except the catheter. When catheter removal is performed, the catheter tip is used to support the diagnosis whenever available.
- CVC-related thrombosis is defined by clinical (eg, swelling, pain, and redness) and radiologic findings.
- Mechanical complications are defined as obstruction, occlusion, breakage, migration, or dislodgment (accidental withdrawal) of the catheter. Ruptures requiring replacement are also counted.

#### Secondary Clinical Outcomes

Postdischarge consultations and rehospitalizations related to CVCs within 30 days were tracked.

#### Primary Educational Outcome

The overall improvement in compliance with the national recommendations of the SF2H following the intervention will be evaluated. Scores will be calculated at the ICU level rather than on an individual basis.

### Statistical Analysis

Descriptive and bivariate analyses will be conducted. Data will be reported as frequencies (%) for categorical data or means (SD) for continuous data. The normality of continuous variables will be checked. Comparisons will use appropriate tests (chi-square, Fisher, *t* test, Mann-Whitney).

Epidemiological, clinical, and microbiological data will be described and interpreted independently. Participants will be categorized into different groups according to their status with respect to end points (ie, complications or not).

Poisson regression will estimate incidence rate ratios and 95% CIs for complication incidence rates. Multivariate regression models (logistic, Cox, or Poisson) will be used to identify factors significantly associated with complications.

The data will be analyzed using the program SPSS (version 21; IBM Corporation).

The complete results are expected in the third quarter of 2025.

## Results

Among the 812 patients admitted to the participating wards as of December 2024, 640 met the inclusion criteria and were enrolled in the study. Data collection is ongoing. Once completed, data entry and verification will be performed, followed by statistical analyses scheduled for the third quarter of 2025.

## Discussion

This study protocol presents a quasi-experimental, prospective before-after design aimed at evaluating the effectiveness of a multifaceted educational intervention to improve adherence to national guidelines for CVC management in ICUs. We anticipate that the implementation of a targeted training program delivered consistently across participating ICUs will lead to measurable improvements in adherence to evidence-based practices and a corresponding reduction in CVC-related complications, such as CLABSI, catheter-related thrombosis, and mechanical issues.

Previous studies have shown that well-structured and context-specific educational programs can bridge the gap between clinical guidelines and bedside practice [8,9,16]. Building on this, our study combines real-time audits and feedback, standardized educational content, and multimodal communication strategies. Unlike single-center initiatives, our study is implemented across 4 ICUs within a large university hospital, providing a broader view of practice variability and revealing the significant deviations from best practices—particularly concerning hand hygiene and flushing technique—highlighting the need for a structured intervention [6].

A recent review supports the view that improving adherence to clinical guidelines necessitates a multifaceted approach addressing both psychological and practical barriers. Effective interventions typically include education and feedback [17].

Despite advancement in clinical protocols, managing CVC-related complications remains a significant challenge. By integrating audits, targeted education, and reminders of best practices, this study aims to reduce practice variability and promote the consistent application of national guidelines, ultimately improving patient outcomes. We hope this initiative will serve as a reproducible model for other institutions seeking to improve care quality and reduce catheter-associated risks.

A key strength of this study is its pragmatic, real-world design. The educational sessions are delivered by the same team using standardized materials across all participating ICUs, ensuring consistent content. Real-time bedside audits further strengthen data reliability by minimizing missing data and allowing objective assessment of clinical practices. Additionally, the protocol includes an extended follow-up period of 30 days per catheter, allowing for comprehensive monitoring for catheter-related complications beyond the ICU setting. This includes complications arising after ward transfer or hospital discharge, providing a more accurate estimation of complication rates.

However, several limitations must be acknowledged. First, no formal sample size or power calculation was performed. The number of patients and audit observations was determined based on feasibility and expected ICU admissions. Approximately 50 audit observations per ICU were planned, covering CVC insertion, maintenance, and removal practices. In addition, a 10-month patient inclusion period was chosen to ensure sufficient volume for robust preintervention and postintervention comparisons. While the absence of formal power estimation is a limitation, the expected sample size and data collection strategy should provide sufficient statistical power to support meaningful analyses and conclusions.

Second, the study excludes patients in palliative care or with hemodynamic instability. This choice was based on both ethical and methodological considerations. Under French research regulations, informed nonopposition is required for participation; in practice, obtaining this from end-of-life or critically unstable patients is often challenging or inappropriate. Furthermore, these patients may follow atypical care pathways that could introduce bias in the evaluation of adherence to standard catheter care protocols. We therefore focused on patients whose clinical condition allowed for the consistent application and assessment of best practices.

Future research could explore the adaptation of this intervention to other hospital departments or high-risk patients. Additional components, such as e-learning modules or behavior change models, may further enhance the intervention’s effectiveness and sustainability.

Results from this study will be disseminated through publication in a peer-reviewed journal and presentations at national and international conferences on infection control and critical care. Feedback will also be shared with ICU teams and hospital leadership to support ongoing quality improvement initiatives.

## Abbreviations

BD: Becton, Dickinson and Company
CLABSI: central line–associated bloodstream infection
CVC: central venous catheter
HCW: health care worker
ICU: intensive care unit
SF2H: French Society of Hospital Hygiene
